# Metabolomics Combined with Transcriptomics Analysis Reveals the Regulation of Flavonoids in the Leaf Color Change of *Acer truncatum* Bunge

**DOI:** 10.3390/ijms252413325

**Published:** 2024-12-12

**Authors:** Yinglun Sun, Ran Yu, Yushan Liu, Jian Liu, Xinyu Zhang, Zaixin Gong, Tongbao Qu

**Affiliations:** College of Forestry and Grassland, Jilin Agricultural University, Changchun 130118, China; sunyinglun123@163.com (Y.S.); yuran77582023@163.com (R.Y.); 18943478100@163.com (Y.L.); liujian13028320@163.com (J.L.); zhangxinyu071@163.com (X.Z.)

**Keywords:** sapindaceae, secondary metabolites, flavonoid and anthocyanin biosynthesis, gene expression, multivariate analysis

## Abstract

The color variation of the leaves in autumn is a significant ornamental feature of *Acer truncatum* Bunge, especially when the leaves gradually become redder. Many studies focused on leaf color changes; however, less research has been conducted on the mechanism by which *A. truncatum*’s autumn leaves turn red. Red, middle and green leaves of *Acer truncatum* were used as the study materials to evaluate their flavonoid-related metabolites and infer gene and metabolite expression patterns in conjunction with transcriptome expression. For a start, phenotypic and leaf color parameters analyses showed that red leaves had the highest color redness and greenness (a*). In addition, a total of 23 flavonoid-related metabolites were identified through the metabolome, including five anthocyanins. Of them, cyanidin 3-O-β-D-sambubioside, cyanidin 3-O rutinoside, pelargonidin 3-O-3″,6″-O-dimalonylglucoside, delphinidin 3,7-di-O-β-D-glucoside and 3-O-β-D-sambubioside would help the leaves turn red in *A. truncatum*. Similarly, combined transcriptomics and metabolomics analyses showed that most genes in the flavonoid and anthocyanin biosynthetic pathways were differentially expressed in both types of leaves. Chalcone synthase (*CHS*), dihydroflavonol 4-reductase (*DFR*) and anthocyanin synthase (*ANS*) could affect flavonoid synthesis during leaf color change. This study could provide data for the genetic improvement of maple plants by exploring valuable metabolites and genes in flavonoid synthesis, and enhance the understanding of different developmental stages.

## 1. Introduction

*Acer truncatum* (Sapindaceae) is one of the most important deciduous maple trees and is often used for its ornamental autumn leaf color in China [1,2,3]. More and more studies are focusing on the mechanism of leaf discoloration in which the leaf color of trees often changes from green to yellow and then to red in autumn [4]. Some studies found that the chloroplast ultrastructure of *Forsythia suspensa* (thunb.) vahl had a looser structure than green leaves, and V-myb avian myeloblastosis viral oncogene homolog (*MYB*), basic helix–loop–helix (*bHLH),* NAM, ATAF and CUC (*NAC*) were associated with the leaf pigment compounds of *Fraxinus angustifolia* vahl [5]. Some studies on maple leaf color changes found that anthocyanin synthase (*ANS*) and bronze-1 (*BZ1*) in *A. mandshuricum* Maxim resulted in the accumulation of cyanidin 3-O-glucoside, which causes a significant reddening of the leaf blade [6]. However, little effort has been made to elucidate the mechanism underlying the variation in color between red, yellow and green leaves of *A. truncatum*.

In general, the color variation of leaves is more complex than that of corollas [7,8,9]. Green leaves are mainly the result of the dominance of chlorophyll content among all pigments [10,11]. By contrast, the formation of yellow leaves is mainly due to the gradual degradation of chlorophyll, resulting in the dominance of carotenoids in the leaves [12]. However, flavonoid biosynthesis is the determining factor for the change of leaf color to a non-yellow color [13]. Flavones, flavonols, flavanones, flavanonols, isoflavones, catechins, anthocyanins and proanthocyanidins belong to subgroups of flavonoids [14,15]. Anthocyanins are particularly the main reason for the red color of the leaves, and this pigment also helps the plant resist various biotic and abiotic stresses [16]. In line with the previous analyses, there are two main functional functions of naturally occurring anthocyanins [17]: one is the resistance to external stresses and the other is using different color strategies to complete the life cycle [18,19]. Consequently, the accurate identification of flavonoids and even anthocyanin compounds in maple is very important for the effective use of forest resources.

Plant metabolomics is the qualitative and quantitative study of small-molecule metabolites in plants that helps researchers better understand patterns of metabolite synthesis and accumulation [20]. Currently, research on plant metabolites is mainly concerned with crop improvement, assisted breeding, discovering biomarkers, the assessment of nutrients, and biotic and abiotic stress studies [21,22,23,24]. Metabolomics for the analysis of flavonoid metabolites is commonly used to analyze the mechanism of plant color formation [7]. For example, rice during yellowing is induced by the metabolism of flavones, flavonols, isoflavones, and anthocyanidins [25]. Furthermore, a significant correlation was found between the accumulation of malvidin 3-O-glucoside and pelargonidin 3-O-glucoside and the change in leaf color from green to red in *Padus virginiana* [26]. These results indicate that metabolomics is an important and effective method for analyzing the mechanisms of plant color formation.

In order to comprehensively analyze the relationship between leaf color and flavonoids and anthocyanins, leaves from the same plant at three developmental stages, namely red, medium and green leaves, were selected as study materials. Metabolomics was used to analyze the changing pattern of flavonoid metabolites and key metabolites in the process of the leaf color change from red to green, and the mechanism of the leaf color difference was explored using transcriptomics analysis. Candidate genes and metabolic pathways for leaf color variation were further demonstrated. Our results provide a new perspective to understand the flavonoid metabolism of *A. truncatum* at different developmental stages, which is conducive to the utilization of its leaf resources.

## 2. Results

### 2.1. Variations in Phenotypes and Analysis of Colour Parameters

**Figure 1:**
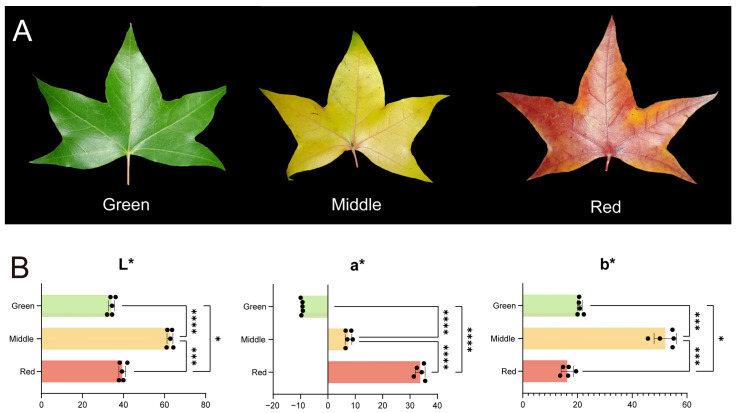
Phenograms of *A. truncatum* and its leaf color parameters. (**A**) Morphological observations of red, middle and green leaves. (**B**) Lab values of leaf color parameters (* *p* < 0.05, *** *p* < 0.001 and **** *p* < 0.0001).

The leaves of *A. truncatum* showed colors at different stages of fall leaf coloration. With the development of the leaves, the leaves gradually changed from green to middle, and finally the mature leaves developed into red, as seen in Figure 1A. During the change of leaf color from green to red, observations showed that the trend of L* and b* values followed the same patterns, with an initial increase and then a decrease, and the overall fluctuation range was large (Figure 1B). However, L* and b* were not significantly different between red and green leaves. Notably, a* showed a continuous upward trend, which was significantly different among all groups.

### 2.2. Analysis of Bioactive Flavonoids

**Figure 2:**
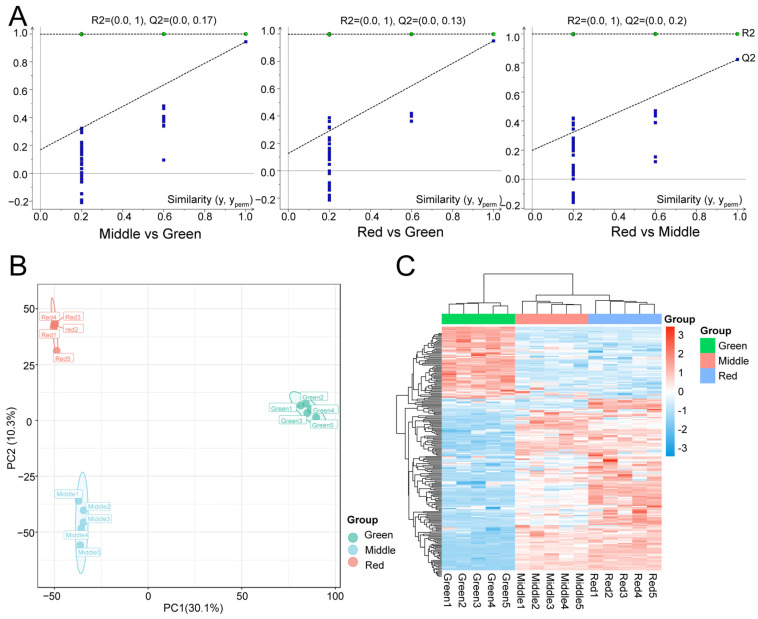
OPLS-DA permutation test plot, PCA score plot and cluster analysis plot for all samples. (**A**) The result is reliable when it is satisfied that both Q2 and R2 are lower than Q2 and R2 in the upper right-hand corner. (**B**) Circles indicate 95% confidence intervals. (**C**) The redder the color, the higher the expression, and the bluer, the lower the expression.

In the OPLS-DA model, the Q2 values for pairwise comparisons exceeded 0.82 and the Q2 values exceeded 0.9 in the two pairwise groups compared with green leaves (Figure 2A). The PCA showed that the composition of the metabolites for the leaf colors of the three different developmental phases differed considerably (Figure 2B). In the clustered heat map containing all samples, leaf metabolites in the red and middle phases had similar expression patterns. The metabolites of the green phase were distinctly different from the metabolites of the other phases and they were assigned between the two branches of the cluster (Figure 2C).

The flavonoids were analyzed in *A. truncatum* (Figure S1 and Table S1). In the flavonoid category, o-methylated flavonoids, flavans, flavonoid glycosides, flavones, and biflavonoids and polyflavonoids were identified. Among the o-methylated flavonoids, hesperetin was identified. Among the flavonoid glycosides, cyanidin 3-glucoside, isoquercitrin, kaempferitrin, peonidin-3-glucoside, pelargonidin 3-sophoroside, myricitrin, quercitrin and astragalin were found. In the flavans class, epicatechin, catechin, naringenin, leucopelargonidin, (-)-epigallocatechin, epigallocatechin gallate and eriodictyol were identified. Among the biflavonoids and polyflavonoids, procyanidin B2 was found. The last remaining six belonged to the flavans.

### 2.3. Analysis of Differentially Expressed Flavonoid Metabolites and KEGG Classification

**Figure 3:**
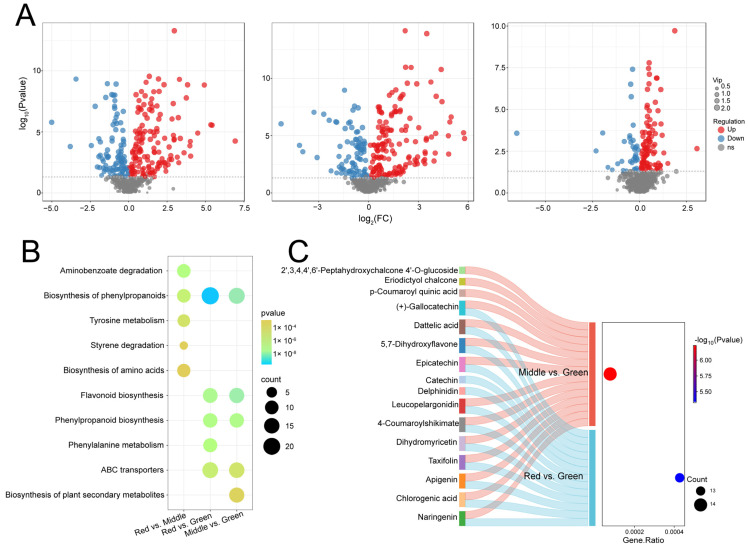
Differential metabolite screening volcano, enrichment bubble and sanger diagrams. (**A**) The red color indicates an up-regulation of metabolite expression in the group and the blue color indicates down-regulation. (**B**) Display of the 5 most significant items in each comparison group. (**C**) Flavonoids in different groups of middle vs. green and red vs. green.

Pairwise comparisons were made between the three groups of materials with different periods of leaf color change (Figure 3A and Table S2). A total of 134 significant differentially expressed metabolites (DEMs) were identified between red and middle leaves, of which 98 increased and 36 decreased. In addition, 243 significant DEMs were identified in the comparison between red and green leaves, of which 158 were increasing and 85 were decreasing. Meanwhile, 237 significant DEMs were identified in middle leaves compared with green leaves, of which 143 increased and 94 decreased.

The KEGG classification showed that the significant DEMs of red and middle color were mainly involved in aminobenzoate degradation, the biosynthesis of phenylpropanoids, central carbon metabolism in cancer, tyrosine metabolism and styrene degradation pathways (Figure 3B). Red and green were mainly related to the biosynthesis of biosynthesis of phenylpropanoids, flavonoid biosynthesis, phenylpropanoid biosynthesis, phenylalanine metabolism and ABC transporters having the smallest *p*-value. The significant DEMs of middle and green were mainly enriched in the biosynthesis of phenylpropanoids, flavonoid biosynthesis, phenylpropanoid biosynthesis, ABC transporters and the biosynthesis of plant secondary metabolites. The significant DEMs of flavonoid biosynthesis in red vs. green and middle vs. green were subsequently analyzed (Figure 3C). In red compared with green, there were 13 flavonoid metabolites of which 5 were significantly up-regulated. In addition, the expression of seven flavonoid metabolites was increased in middle compared to green. Notably, most flavonoid metabolites, especially naringenin, chlorogenic acid, apigenin, taxifolin, dihydromyricetin, 4-coumaroylshikimate, leucopelargonidin, 5,7-dihydroxyflavone, dattelic acid and (+)-gallocatechin, were expressed in both comparison groups.

### 2.4. Analysis of Transcriptome Results and Functional Annotation

**Figure 4:**
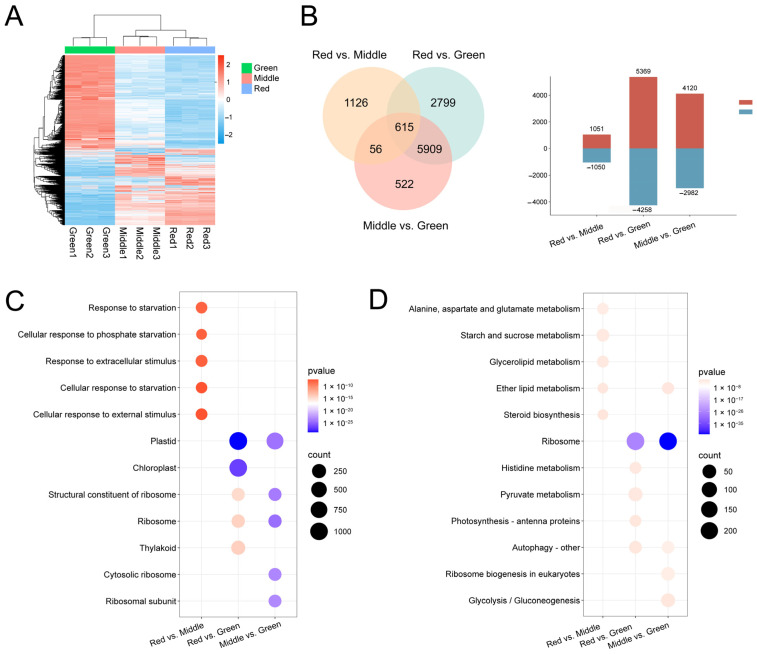
Clustered heatmaps, Venn plots and the GO and KEGG enrichment in the middle vs. green, red vs. green and red vs. middle comparisons. (**A**) The redder the color, the higher the expression, and the bluer, the lower the expression. (**B**) Overlapping regions indicate shared differential genes between comparison groups; red in the bar indicates up-regulation and blue indicates down-regulation. (**C**) GO-enriched bubble charts, with the five most significant terms selected for each comparison group. (**D**) KEGG-enriched bubble charts, with the five most significant pathways selected for each comparison group.

In the clustered heat map containing all samples, genes in the red and middle phases had similar expression patterns (Figure 4A). The genes of the green phase were distinctly different from the metabolites of the other phases and they were assigned between the two branches of the cluster. The differentially expressed genes (DEGs) of red, middle and green leaves in different developmental stages were analyzed (Figure 4B). A total of 2101 DEGs were obtained from the red vs. middle comparison, and the higher number of 1051 DEGs was upregulated. In addition, the highest number of DEGs was 9627 in red vs. green, among which 5369 genes showed upregulated expression, and 4258 genes showed downregulated expression. Simultaneously, the number of 7102 DEGs was obtained in middle vs. green, in which the genes with downregulated expression accounted for 41.98% of all DEGs.

The gene ontology (GO) enrichment results showed that the DEGs of the red vs. middle group were significantly enriched in biological processes such as response to starvation, cellular response to phosphate starvation, response to extracellular stimulus, cellular response to starvation and cellular response to external stimulus (Figure 4C). The DEGs of red vs. green were enriched in plastid, chloroplast, structural constituent of ribosome, ribosome and thylakoid. The DEGs of the red vs. green group were enriched in chloroplast. In addition, 37 of these genes were annotated to the porphyrin metabolism pathway (Table S3 and Figure S2). The Kyoto encyclopedia of genes and genomes (KEGG) enrichment results showed that the red vs. middle group was annotated into 116 metabolic pathways (Figure 4D), where alanine, aspartate and glutamate metabolism, starch and sucrose metabolism, glycerolipid metabolism, ether lipid metabolism and steroid biosynthesis pathways were significantly enriched. The red vs. green were enriched in 130 metabolic pathways and the middle vs. green were enriched in 128 metabolic pathways.

### 2.5. Flavonoid Biosynthesis in Relation to Genes and Metabolites

**Figure 5:**
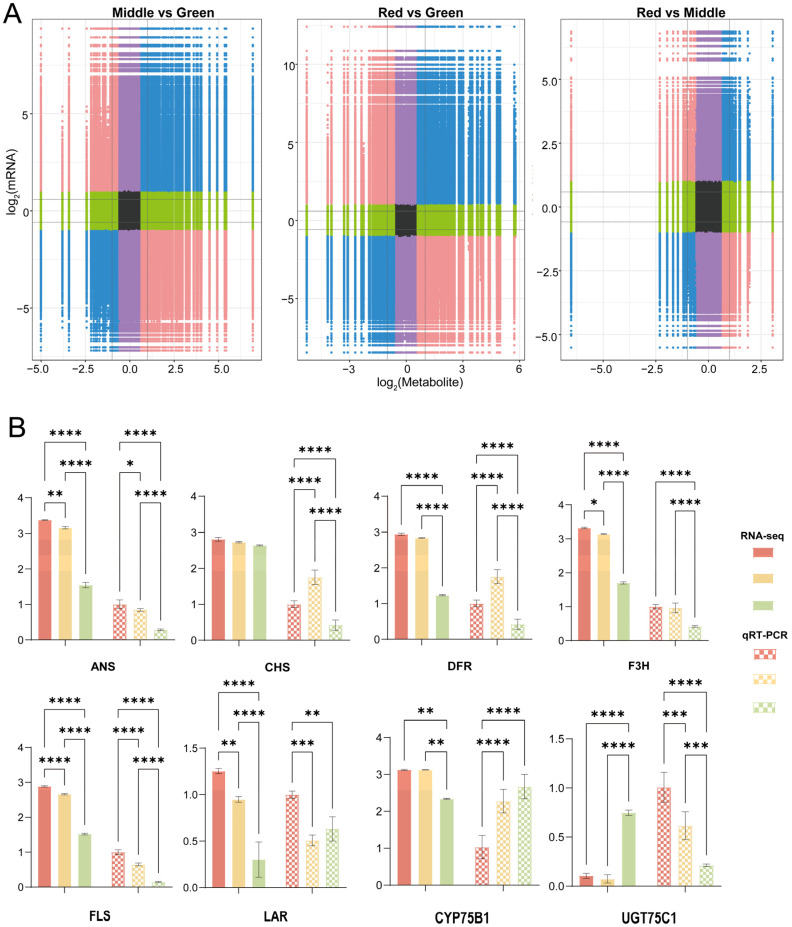
The 9-quadrant diagram and the qRT-PCR results. (**A**) The 9-quadrant diagram based on the correlation analysis of metabolites and genes in the middle vs. green, red vs. green and red vs. middle comparisons. Red dots show opposite changes in metabolism and mRNAs, blue dots show opposite changes in metabolism and mRNAs, green dots show differences in metabolism only, purple dots show differences in mRNAs only and black dots show no differences in metabolism and mRNAs. (**B**) Eight gene expression patterns in different leaf colors in qRT-PCR. (* *p* < 0.05, ** *p* < 0.01, *** *p* < 0.001 and **** *p* < 0.0001).

The nine-quadrant plot based on correlation analysis shows that metabolite and gene expression patterns are consistent in quadrants three and seven (Figure 5A). We found that kaempferin, proanthocyanidin B2, 5,7-dihydroxyflavone, apigenin, quercetin, epigallocatechin gallate and astragalin were identified as being related the six genes *F3H*, *FLS*, *ANS*, *LAR*, *DFR, CHS* and *CYP75B1*. The epicatechin, naringenin, leucopelargonidin and *UGT75C1* genes were identified as related.

To further confirm the reliability of the RNA-Seq results, these eight candidate genes were selected for verification. The qRT-PCR analysis of the genes encoding these enzymes showed that all genes were significantly down-regulated (Figure 5B). A comprehensive analysis of enzyme activity and the corresponding gene expression pattern showed that the changing trend of enzyme activity of *ANS*, *CHS*, *DFR*, *FLS* and *LAR* was consistent with that of the gene expression pattern, while that of the other two enzymes was different from that of the gene expression pattern.

### 2.6. Analysis of Flavonoid and Anthocyanin Biosynthesis

**Figure 6:**
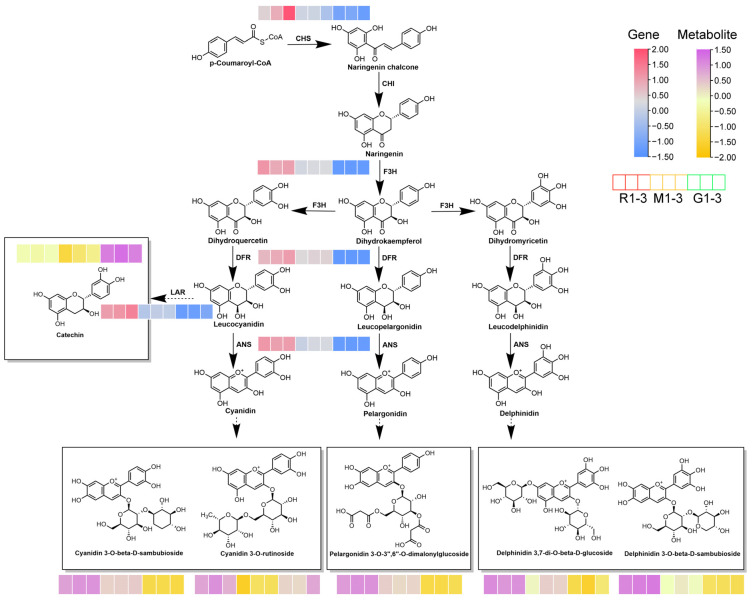
Combined analysis of metabolites and genes involved in flavonoid and anthocyanin biosynthesis. Pink and green heatmaps indicate metabolite accumulation. Blue and red heatmaps show gene expression. CHS, chalcone synthase; CHI, chalcone isomerase; F3H, flavanone 3-hydroxylase; DFR, dihydroflavonol 4-reductase; ANS, anthocyanidin synthase; LAR, Leucoanthocyantin reductase.

The combination analysis indicated that the expression of genes related to flavonoid synthesis in red leaves was higher than that in green leaves in the same developmental stage (Figure 6). Under the action of *DFR*, dihydroquercetin (DHQ), dihydrokempferol (DHK) and dihydromyricetin (DHM) were transformed into leucocyanidin, leucopelargonidin and eucodelphinidin. They were generated as anthocyanins under the action of *ANS*, so anthocyanins accumulate in red and middle leaves. With the development of leaf color, the *CHS*, *F3H*, *DFR* and *ANS* genes were continuously upregulated in the subsequent developmental stages. By contrast, *LAR* showed continuous downregulation, which led to the reduced conversion of leucocyanidin into catechin. Five relevant metabolites, namely cyanidin 3-O-β-D-sambubioside, cyanidin 3-O rutinoside, pelargonidin 3-O-3″,6″-O-dimalonylglucoside, delphinidin 3,7-di-O-β-D-glucoside and 3-O-β-D-sambubioside, showed differential changes in the process. In addition, compared with green leaves, cyanidin 3-O-beta-D-sambubioside and cyanidin 3-O rutinoside showed the most significant increases in red leaves during the two stages.

### 2.7. Analysis of Proteins Encoding Genes for Flavonoids and Anthocyanin Metabolites

The predicted secondary structures of the eight proteins encoding genes showed that all the proteins consisted of four parts: an alpha helix, an extended chain, a beta turn, and a random coil (Table S4). For another, these proteins had the higher proportion of random coil or α-helix, followed by an alpha helix and an extended chain, and the secondary structure of CYP75B1 had the lowest proportion of β-turn. Second, their encoded proteins’ tertiary structure includes alpha helices, extended chains, and random coils (Figure S3). The tertiary structure shows that the secondary structure further folds in a more regular manner, with similar structures formed by different proteins, indicating that their functions are different and further demonstrating the diversity of functions of its members.

These genes encode a minimum of 265 amino acids and a maximum of 517 amino acids (CYP75B1) (Table S5). Meanwhile, three identical structural domains with two identical motifs were found in F3H, FLS and ANS. The three proteins were predicted to belong to the protein family Plant 2OG-oxidoreductases (2ODOs). CHS possesses the active site of the enzyme chalcone/stilbene synthase. Furthermore, the protein encoded by *CYP75B1* belongs to the cytochrome P450. In addition, the structural genes were usually controlled by transcription factors in leaf color-related biosynthetic pathways, such as *MYB*, *bHLH*, *NAC* and *WRKY* (Figure S4). Our study identified 36 *MYBs*, 126 *bHLHs*, 78 *NACs* and 41 *WRKYs* that were up-regulated in the group of red vs. green. These up-regulated transcription factors were positively correlated with anthocyanin metabolism in leaves. One *NAC* was annotated to the flavonoid synthesis pathway, while two *MYBs* were localized to the porphyrin metabolism pathway (Table S6).

## 3. Discussion

The regulation of plant leaf color is a complex process [27]. Some results showed that anthocyanins were a major class of compounds that cause changes in leaf color in plants [28]. The color of the leaves was in turn related to the content of the mostly studied phytochemicals, which include flavonoids and phenolic acids [29,30,31,32]. Furthermore, fluctuations in plant leaf chemical levels could trigger leaves to take on different colors. The other results showed that leaf pigment content affects leaf color differences and its content was negatively correlated with L* [33]. Furthermore, when the anthocyanin content of *Lycium barbarum* L. accumulated, it was reflected in the color by the process of turning from green to red and continuing to deepen [34]. In this study, L* was assigned the highest value in the middle color, which indicated that the chlorophyll content might reach its lowest at this point. When the leaf color changed from green to red, the chlorophyll content had a tendency to decrease and then increase. It is known that the a* value is positively correlated with anthocyanin; when a* gradually increased, the leaf undergoes the process of anthocyanin accumulation. Therefore, we assumed that the content of both flavonoids and anthocyanins increase gradually in the process of coloration of the leaves to red.

The molecular basis of flavonoid biosynthesis is increasingly important today [35,36]. For instance, the metabolite analysis of *Ficus carica* L. detected 15 different flavonoid-related metabolites, which included the very significant accumulation of the colorless flavonoids procyanidin B1, luteolin-3′,7-di-O-glucoside, epicatechin and quercetin-3-O-rhamnoside in the mature purple peel [37]. A total of 40 flavonoid metabolites were identified through the metabolite extraction and characterization of *Cucumis melo*. In these metabolites, flavonoids, flavanones, isoflavones and anthocyanins were the substances that mainly affected fruit color [38]. The quantitative analysis revealed that the four varieties in question contained a combination of 125 distinct flavonoids in *Actinidia arguta*, with only delphinidin 3-O-glucoside, cyanidin O-octanoic acid and pelargonidin 3-O-β-D-glucoside being detected in the red and purple fruits [39]. An aggregate of 23 flavonoid-related metabolites were detected in this article. These belong to o-methylated flavonoids, flavones, flavans, flavonoid glycosides and biflavonoids and polyflavonoids. However, in this study, only five anthocyanins had significant discrepancies among the different stages: cyanidin 3-O-β-D-sambubioside, cyanidin 3-O rutinoside, pelargonidin 3-O-3″,6″-O-dimalonylglucoside, delphinidin 3,7-di-O-β-D-glucoside and 3-O-beta-D-sambubioside. Interestingly, more anthocyanins associated with the leaf color difference were detected. Additionally, there were similarities and differences in the types of flavonoid metabolites between the above plants and *A. truncatum*. Both it and *Actinidia arguta* also contained delphinidin, which was not present in the other plants, suggesting differences in flavonoid composition between species.

Phenylalanine, an upstream reaction of flavonoids and anthocyanins, was first converted to p-coumaroyl-CoA coenzyme a catalyzed by *PAL*, *C4H* and *4CL* [40]. In the presence of *CHS* and *CHI*, p-coumaroyl-CoA was converted to naringenin chalcone and then to naringenin. The expression of *CHS* was up-regulated in the process of the leaf color change, which was consistent with the qRT-PCR results. Meanwhile, *CHS* was the first key enzyme in flavonoid synthesis and its activity determines the formation of related metabolites [41,42]. The expression of *CHS* in the red leaf stage was highly significant for the green leaf stage, suggesting that *CHS* may be involved in the process of leaf color changes. Then, naringenin was catalyzed by *F3H* to form DHK, which then continued to be catalyzed by *F3H* to form DHQ and DHM, respectively. Under the catalytic action of *DFR* and *ANS*, DHK, DHQ and DHM formed unstable anthocyanins such as cyanidin, pelargonidin and delphinidin, respectively. DHK, DHQ and DHM catalyzed the *DFR* to produce different ones in different plants and the *ANS* was necessary for the accumulation of different anthocyanins [43,44,45]. The unstable anthocyanins produced eventually formed stable anthocyanins in the presence of *UGT75C1*. To summarize, the five stabilities of anthocyanins that were described in detail in the previous paragraph were predominantly found in *A. truncatum*.

2ODDs are a family of proteins with both DIOX_N and 2OG-FeII_Oxy conserved structures [46]. The related article pointed to them as the second-largest family of oxidative enzymes in plants, involved in various oxidative reactions [47]. They are widely involved in secondary metabolic processes in plants, such as the biosynthesis of flavonoids, alkaloids and terpenoids [48,49]. We expected that 2ODD could catalyze the conversion of naringenin into dihydrokaempferol, indicating that the enzyme was a typical F3H [50]. This was demonstrated in the investigation that we have carried out. In our study, F3H, FLS and ANS belonged to this family. They possessed the same conserved structural domains DIOX_N and 2OG-FeII_Oxy described above, which could be involved in flavonoid formation and have an impact on anthocyanin biosynthesis. Correspondingly, we need to focus on the cytochrome P450 family. The P450s are thioredoxin proteins involved in the oxidative degradation of various compounds. P450 was imprinted in *Scutellaria baicalensis* for anthocyanin modification [51]. The characterization and analysis of P450 from grapes revealed that its subfamily CYP75 is involved in anthocyanin production in a similar way [52]. CYP75B1, which undoubtedly possesses the conserved structural domain of P450, affects the production of DHQ, DHK and DHM from naringenin. Increased gene activity of these members from different families triggers the accumulation of flavonoids and anthocyanins in red leaves.

## 4. Materials and Methods

### 4.1. Plant Materials and Sampling

*A. truncatum* growing in the wild on Jilin Agricultural University campus (43°05′–45°15′ N, 124°18′–127°05′ E) was used. Based on the color pattern of the leaves, three different colored leaves of the same plant were selected as study material (red, middle and green leaves). Ten leaves were collected from each group and leaves with a similar location and color on the branches were selected. The test materials were divided into two parts: one half of the samples was quickly scanned for leaf color parameters, and the other half of the samples was quickly fixed with liquid nitrogen and later moved to storage at −80 °C.

### 4.2. Determination of Leaf Colour Parameters

Leaf color was quantified in accordance with the International Commission on Illumination (CII) color standard. Luminosity (L*), a*, and b* values were obtained through the use of a CR30 colorimeter (CHNSpec, Hanghzhou China). After calibration using the colorimetric plate, five points were randomly selected and averaged on each blade, with the objective of avoiding the leaf edges and radial main veins [53,54]. The exercise was repeated five times for each leaf color.

The meanings of the three parameters are as follows. L* indicates the brightness of the color, where a positive number means whitish and a negative number means blackish. Additionally, a* indicates the red–green value, where a positive value means the color is redder and a negative value means it is greener. Finally, b* indicates the yellow–blue value, where positive values are yellowish and negative values are bluish.

### 4.3. Extraction of Total Metabolites

First, the sample was weighed accurately in a 2 mL centrifuge tube and 600 µL of MeOH containing 2-Amino-3-(2-chloro-phenyl)-propionic acid (4 ppm) was added and vortexed for 30 s. Second, The sample was then placed in a tissue grinder and ground at 55 Hz for 60 s, followed by sonication at room temperature for 15 min. Finally, the sample was centrifuged at 12,000 rpm at 4 °C for 10 min on a H1850-R refrigerated centrifuge (Cence, Changsha, China), and the supernatant was taken through a 0.22 μm membrane and added into the detection vial for LC-MS detection [55].

### 4.4. Metabolomics Analysis

The LC analysis was performed on a Vanquish UHPLC System (Thermo Fisher Scientific, Waltham, MA, USA). Chromatography was carried out with an ACQUITY UPLC ^®^ HSS T3 (2.1 × 100 mm, 1.8 µm) (Waters, Milford, MA, USA). The column was maintained at 40 °C. The flow rate and injection volume were set at 0.3 mL/min and 2 μL, respectively.

Mass spectrometric detection of metabolites was performed on Q Exactive (Thermo Fisher Scientific, Waltham, MA, USA) with an ESI ion source. Simultaneous MS1 and MS/MS (full MS-ddMS2 mode, data-dependent MS/MS) acquisition was used. The parameters were as follows: capillary temperature, 325 °C; MS1 range, *m*/*z* 100–1000; MS1 resolving power, 70,000 FWHM; number of data-dependent scans per cycle, 10; MS/MS resolving power, FWHM; normalized collision energy, 30 eV; dynamic exclusion time, automatic.

The raw mass spectrometry downcomer files were converted into the mzXML file format using the MSConvert tool in the Proteowizard software package (v3.0.8789). Peak detection, peak filtering and peak alignment were carried out using the R XCMS software package (v3.12.0), and a list of substances for quantification was obtained. Substances with coefficients of variation smaller than 30% in the QC samples were then retained for subsequent analysis.

The molecular weight of the metabolite was ascertained by means of the mass-to-charge ratio of the parent ion present in the primary mass spectrum. Furthermore, the molecular formula was deduced on the basis of the mass number deviation, as well as the information provided by the additional ions. Following this, the metabolite was matched with a previously existing record in a database, thereby achieving its preliminary identification. Meanwhile, the metabolites detected in the secondary spectrum were subjected to a process of secondary identification. This involved the matching of the metabolite data with the information contained in the database, including the fragment ions of each metabolite.

### 4.5. Total RNA Extraction and Transcriptome Sequencing

The mRNA with polyA structure in the total RNA was enriched by Oligo(dT) magnetic beads. Subsequently, the RNA was subjected to ionic interruption, which fragmented it into fragments of approximately 300 base pairs in length. The initial cDNA strand was synthesized using RNA as a template with a 6-base random primer and reverse transcriptase. The second cDNA strand was then synthesized with the initial cDNA strand serving as the template. Following the construction of the library, the library fragments were amplified by PCR. The library size was 450 bp, and the total and effective concentrations were subsequently determined by an Agilent 2100 Bioanalyzer (Agilent, Santa Clara, CA, USA). Subsequently, the mixed libraries were uniformly diluted to 2 nM and denatured by alkaline denaturation, thereby forming single-stranded libraries. Following the extraction, purification and construction of the libraries, the libraries were subjected to paired-end sequencing using next-generation sequencing (NCBI project: PRJNA1196921).

### 4.6. Identification and Analysis of DEGs

FPKM was used to normalize the raw gene expression calculation. In order to identify genes that were differentially expressed among the three different groups, we identified genes with |log_2_foldchang| > 1 and a *p*-value < 0.05 as DEGs. GO functional enrichment analysis and KEGG pathway analysis were performed on the confirmed DEGs.

### 4.7. qRT-PCR Analysis

Primers specific to structural genes involved in flavonoid biosynthesis were designed for qRT-PCR analysis using Primer Premier 5 software (Table S7). All samples were subjected to three replicates, as were three technical replicates. The internal control genes employed were *actin* and *β-tubulin*. The relative expression levels of the target genes were calculated employing the 2^−∆∆Ct^ methodology. Three experimental replicates were performed for each sample.

### 4.8. Protein Biology Analysis

The secondary structure of the protein encoding the key enzyme gene for flavonoids and anthocyanin synthesis was predicted using the SOPMA online tool (https://npsa.lyon.inserm.fr/cgi-bin/npsa_automat.pl?page=/NPSA/npsa_sopma.html, accessed on 4 October 2024) and modeled for the tertiary structure using the Phyre2 online tool (http://www.sbg.bio.ic.ac.uk/phyre2/, accessed on 4 October 2024) for tertiary structure modeling. The proteins were also functionally analyzed using the website InterPro (https://www.ebi.ac.uk/interpro/, accessed on 5 October 2024).

## 5. Conclusions

In this study, we focused on the diversity of flavonoid compounds in the red, middle and green leaves of *A. truncatum* to explore the molecular mechanisms of leaf color formation. The visual diversity of different leaf colors was first described through leaf color parameters, using a digital method. Moreover, a total of 23 different modified flavonoids were detected by metabolomics. In particular, cyanidin 3-O-β-D-sambubioside, cyanidin 3-O rutinoside, pelargonidin 3-O-3″,6″-O-dimalonylglucoside, delphinidin 3,7-di-O-β-D-glucoside and 3-O-β-D-sambubioside could be responsible for the differences between green and red leaves. Furthermore, RNA-seq analysis showed that the up-regulation of *CHS*, *DFR* and *ANS* expression led to an increase in the corresponding anthocyanin red color, which resulted in the reddening of the leaves. Additionally, *UGT75C1* was correspondingly essential as a downstream gene for the synthesis of anthocyanin. By modeling the proteins encoded by these genes and analyzing the conserved structural domains, the results corroborated the reliability of the metabolomic and transcriptomic data. Overall, the study offers valuable insights into the flavonoid-related metabolite composition in A. truncatum, providing essential reference points for breeders seeking to enhance the pigmentation of this species.

## Data Availability

Data are available upon request.

## Supplementary Materials

The following supporting information can be downloaded at: https://www.mdpi.com/article/10.3390/ijms252413325/s1.
