# Supplementary material for: Metabolomics Combined with Transcriptomics Analysis Reveals the Regulation of Flavonoids in the Leaf Color Change of Acer truncatum Bunge
Source: Int J Mol Sci. 2024 Dec 12;25(24):13325. doi: 10.3390/ijms252413325 (PMC11678339; doi:10.3390/ijms252413325)
Supplement: Supplementary file 1 [file ijms-25-13325-s001.zip › ijms-3327714-supplementary.pdf]

Metabolomics combined with Transcriptomics Analysis Reveals the Regulation of Flavonoids in the Leaf Color Change of *Acer truncatum* Bunge

Supplemental Materials

Figures

Figure S1. Heatmap of clustering of expression of bioactive flavonoids with differences.

Figure S2. Heatmap of gene expression in the porphyrin metabolic pathway.

Figure S3. Predicted tertiary structure of the protein encoding the key enzyme gene for flavonoid synthesis in *A. truncatum*.

Figure S4. The transcription factor in the comparative group red vs. green.

Tables

Table S1. Characterisation of flavonoid metabolomics.

Table S2. Quantity and classification of secondary difference metabolites.

Table S3. Genes annotated to the porphyrin metabolic pathway.

Table S4. The secondary structure of proteins encoded by candidate genes.

Table S5. Structural domains of proteins encoded by candidate genes.

Table S6. Transcription factors affecting leaf colour.

Table S7. The specific primers for the candidate and reference genes for qRT-PCR.

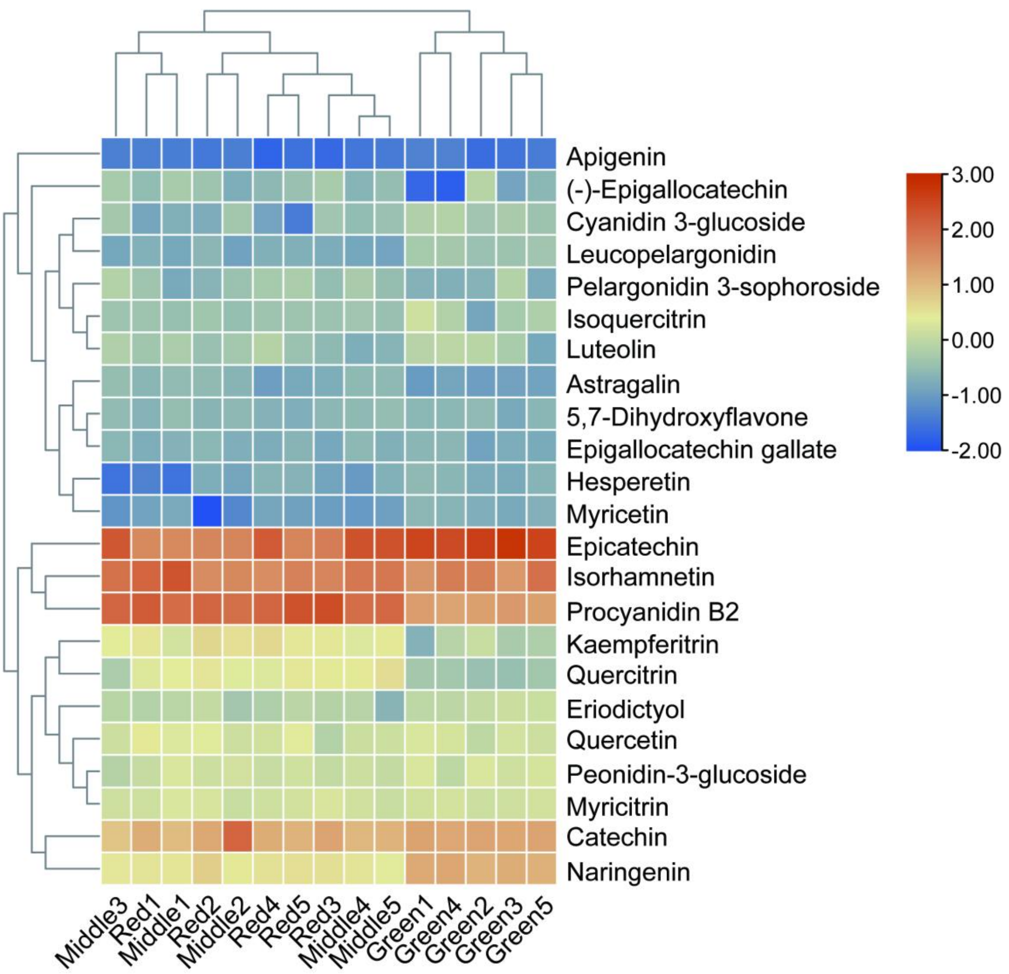

Figure S1. Heatmap of clustering of expression of bioactive flavonoids with differences

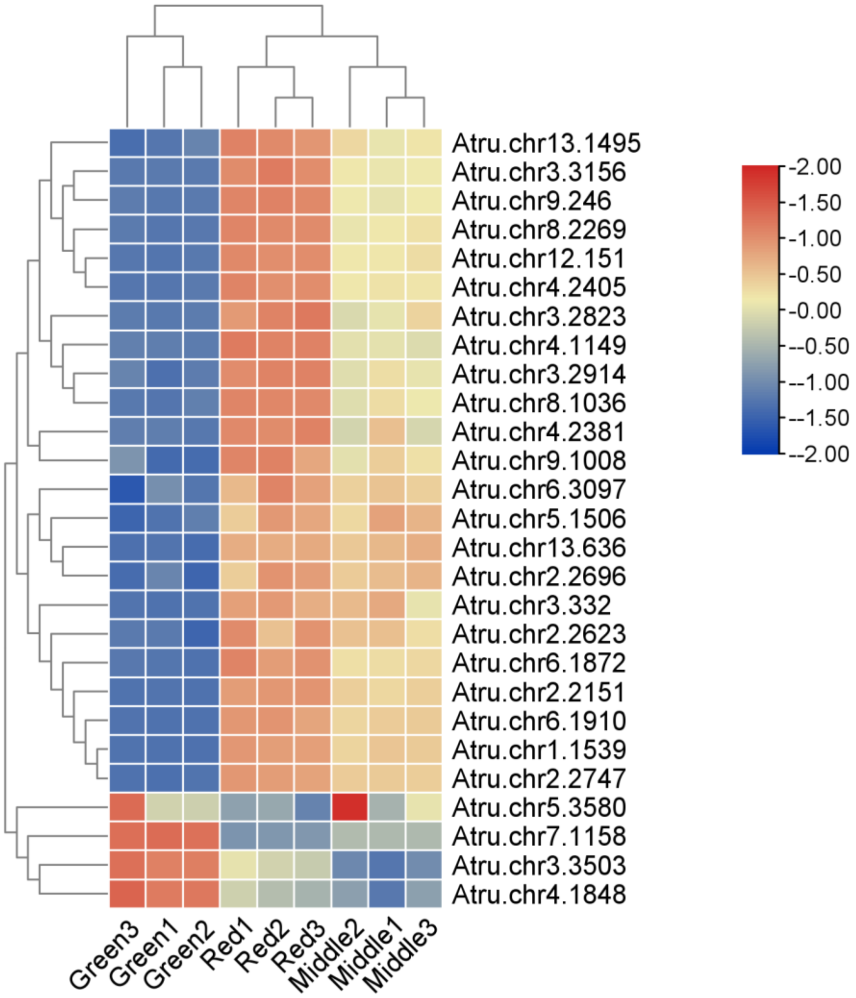

Figure S2. Heatmap of gene expression in the porphyrin metabolic pathway.

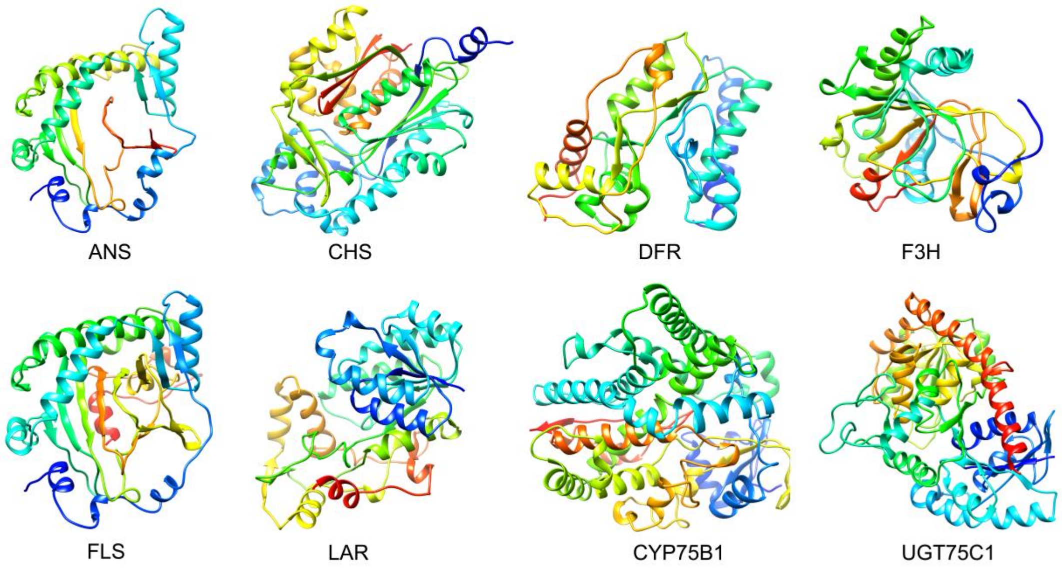

Figure S3. Predicted tertiary structure of the protein encoding the key enzyme gene for flavonoid synthesis in *A. truncatum*.

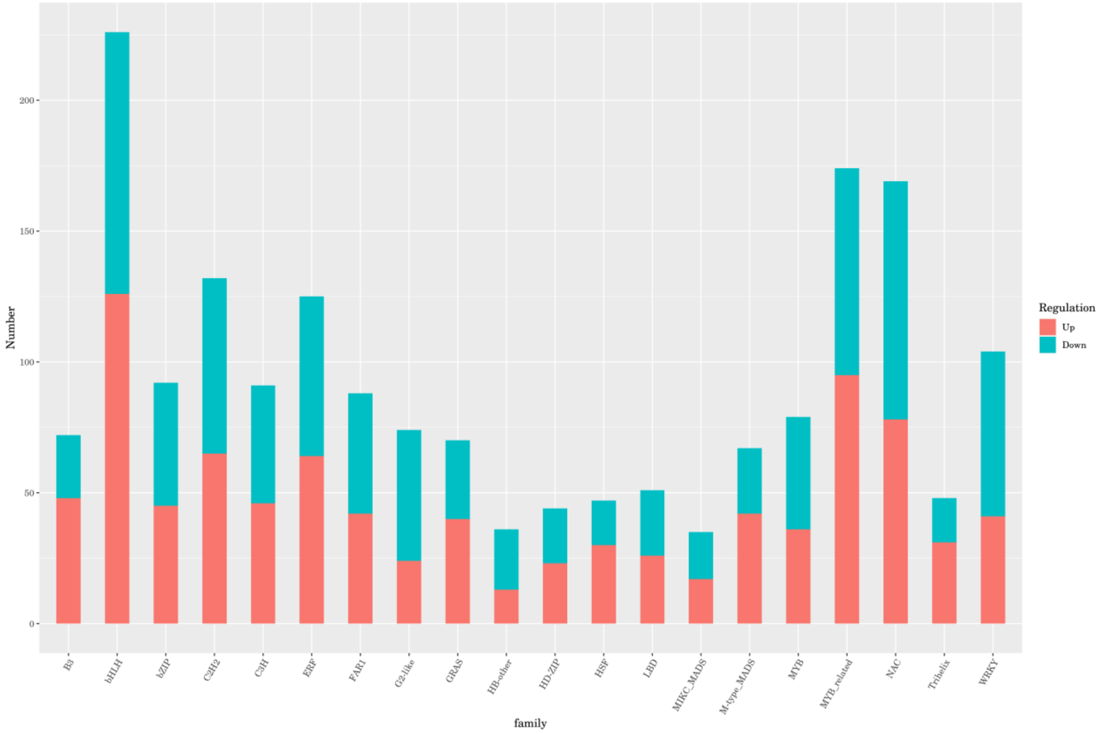

Figure S4. The transcription factor in the comparative group red vs. green.

Table S1. Characterisation of flavonoid metabolomics.

Name	M/Z	Retention time	Ppm	Formula	Precursor ions	Sub class
Hesperetin	303.0864	165.7	0.409124263	$C_{16}H_{14}O_6$	$[M+H]^+$	O-methylated flavonoids
Epicatechin	291.086	257.7	0.948173392	$C_{15}H_{14}O_6$	$[M+H]^+$	Flavans
Cyanidin 3-glucoside	449.1069	480.1	1.579684817	$C21H_{21}O_{11}$	$[M]^+$	Flavonoid glycosides
Catechin	291.0865	275.9	0.769530707	$C_{15}H_{14}O_6$	$[M+H]^+$	Flavans
Isoquercitrin	465.1029	242.8	0.266607669	$C_{21}H_{20}O_{12}$	$[M+H]^+$	Flavonoid glycosides
Naringenin	273.0755	281.8	1.010709493	$C_{15}H_{12}O_5$	$[M+H]^+$	Flavans
Myricetin	319.0449	244.3	0.075224522	$C_{15}H_{10}O_8$	$[M+H]^+$	Flavones
Isorhamnetin	317.0668	309.8	3.860385256	$C_{16}H_{12}O_7$	$[M+H]^+$	Flavones
Kaempferitrin	579.1638	279.8	12.21761443	$C_{27}H_{30}O_{14}$	$[M+H]^+$	Flavonoid glycosides
Leucopelargonidin	291.092	295.8	18.68027417	$C_{15}H_{14}O_6$	$[M+H]^+$	Flavans
Peonidin-3-glucoside	463.119	305.2	8.960147301	$C_{22}H_{23}O_{11}$	$[M+H]^+$	Flavonoid glycosides
Procyanidin B2	579.1475	244.7	2.258324254	$C_{30}H_{26}O_{12}$	$[M+H]^+$	Biflavonoids and polyflavonoids
Pelargonidin 3-sophoroside	595.1407	165.3	5.668875874	$C_{27}H_{31}O_{15}$	$[M]^+$	Flavonoid glycosides
5,7-Dihydroxyflavone	253.0494	429.4	3.503944479	$C_{15}H_{10}O_4$	$[M-H]^-$	Flavones
Apigenin	269.0454	435.9	0.282567455	$C_{15}H_{10}O_5$	$[M-H]^-$	Flavones
(-)-Epigallocatechin	305.0662	217.7	1.486743146	$C_{15}H_{14}O_7$	$[M-H]^-$	Flavans
Quercetin	301.0355	367.4	0.252461919	$C_{15}H_{10}O_7$	$[M-H]^-$	Flavones
Luteolin	285.0404	374.3	0.084198591	$C_{15}H_{10}O_6$	$[M-H]^-$	Flavones
Myricitrin	463.0897	451.1	3.187287474	$C_{21}H_{20}O_{12}$	$[M-H]^-$	Flavonoid glycosides
Quercitrin	447.0929	451.5	0.894668647	$C_{21}H_{20}O_{11}$	$[M-H]^-$	Flavonoid glycosides
Epigallocatechin gallate	457.0763	260.2	2.844164093	$C_{22}H_{18}O_{11}$	$[M-H]^-$	Flavans
Astragalin	429.082	336.9	0.489416941	$C_{21}H_{20}O_{11}$	$[M-H_2O-H]^-$	Flavonoid glycosides
Eriodictyol	287.056	341.1	0.431971462	$C_{15}H_{12}O_6$	$[M-H]^-$	Flavans

Table S2. Quantity and classification of secondary difference metabolites.

Classification	Count	Classification	Count	Classification	Count
Benzene and substituted derivatives	65	Keto acids and derivatives	4	Aromatic hydrocarbons	1
Carboxylic acids and derivatives	56	Phenylpropanoic acids	4	Benzodiazepines	1
Fatty Acyls	45	Purine nucleosides	4	Biotin and derivatives	1
Organooxygen compounds	43	Quinolines and derivatives	4	Cinnamaldehydes	1
Prenol lipids	34	5'-deoxyribonucleosides	3	Dioxanes	1
Flavonoids	23	Azoles	3	Furans	1
Steroids and steroid derivatives	20	Carbohydrates and carbohydrate conjugates	3	Glycerophospholipids	1
Phenols	19	Isoflavonoids	3	Lactones	1
Organic oxides	11	Pyrimidine nucleosides	3	Linear 1,3-diarylpropanoids	1
Cinnamic acids and derivatives	9	Alcohols and polyols	2	Lipids and lipid-like molecules	1
Endogenous Metabolites	8	Benzodioxoles	2	Naphthalenes	1
Indoles and derivatives	8	Benzopyrans	2	Polycyclic hydrocarbons	1
Imidazopyrimidines	7	Benzothiazines	2	Pteridines and derivatives	1
Organonitrogen compounds	6	Epoxides	2	Pyrans	1
Azacyclic compounds	5	Hydroxy acids and derivatives	2	Pyrroles	1
Coumarins and derivatives	5	Organic phosphonic acids and derivatives	2	Tetrapyrroles and derivatives	1
Pyridines and derivatives	5	Alkyl halides	1		
Diazines	4	Amines	1		

Table S3. Genes annotated to the porphyrin metabolic pathway.

ID	FoldChange (Green/Red)	Pvalue	Annotation
Atru.chr1.1539	24.54845127	0	Magnesium-protoporphyrin IX
Atru.chr7.1158	0.051298909	0	Heme oxygenase 1
Atru.chr2.2747	72.50183172	5.6884E-249	Protochlorophyllide reductase
Atru.chr6.1910	11.37693585	3.8923E-197	Magnesium protoporphyrin IX methyltransferase
Atru.chr4.1848	0.301149606	1.3795E-182	7-hydroxymethyl chlorophyll a reductase
Atru.chr3.3503	0.345140556	5.4271E-150	Pheophorbide a oxygenase
Atru.chr8.2269	5.475319209	3.2751E-138	Chlorophyll synthase
Atru.chr4.1149	4.362371423	2.3369E-136	Ferrochelatase-2
Atru.chr4.2405	5.290641524	4.958E-130	Chlorophyllase-2
Atru.chr9.246	3.064252604	2.7399E-128	Geranylgeranyl diphosphate reductase
Atru.chr6.1872	3.317793731	1.81287E-98	Glutamate-1-semialdehyde 2, 1-aminomutase
Atru.chr12.151	4.046382907	1.73888E-93	Uroporphyrinogen decarboxylase
Atru.chr2.2151	7.034136221	1.5344E-77	Uroporphyrinogen decarboxylase 1
Atru.chr3.3156	7.527625449	9.46313E-72	Phytochromobilin:ferredoxin oxidoreductase
Atru.chr4.2381	4.178207582	8.11141E-53	Chlorophyllase-2
Atru.chr8.1036	3.13467733	2.9683E-34	Oxygen-dependent coproporphyrinogen-III oxidase
Atru.chr13.636	18.41044443	1.88978E-29	Probable inactive heme oxygenase 2
Atru.chr3.2823	3.254333167	5.66911E-22	Phytochromobilin:ferredoxin oxidoreductase
Atru.chr13.1495	2.276040686	1.8788E-20	Delta-aminolevulinic acid dehydratase 1
Atru.chr5.3580	0.412477378	1.11352E-19	Glutamate--tRNA ligase
Atru.chr2.2623	6.273207756	2.783E-16	Cytochrome c oxidase assembly protein COX15
Atru.chr9.1008	11.47179477	1.86995E-13	Heme chaperone
Atru.chr3.2914	2.890046089	4.6476E-11	Geranylgeranyl diphosphate reductase
Atru.chr3.332	9.503765212	3.02832E-08	Red chlorophyll catabolite reductase
Atru.chr2.2696	3.216703777	2.24247E-06	Cytochrome c oxidase assembly protein COX15
Atru.chr6.3097	4.269995132	2.13402E-05	Frataxin
Atru.chr5.1506	2.063961803	0.000136161	Chlorophyllase-1

Table S4. The specific primers for the candidate and reference genes for qRT-PCR.

Name	ID	top	bottom
F3H	Atru.chr3.799	TCGTCTCCAGCCATCTTCAG	CCTCCTTCTCCAACCCCATT
FLS	Atru.chr13.1396	CTACTGGAGTTGCTGTCGGA	AAGTATGGTGAGTGCGGACA
ANS	Atru.chr9.1498	AATGATCAGGCTTCGGGGAA	AAGCTTCTCAGTTGCCTTGC
LAR	Atru.chr11.1351	CCAGGGCTGAGGATGTACAA	TCCCAGCTACAAAGTACGCT
DFR	Atru.chr13.438	CACATCATCAGCAGGAACCG	GACGGCATGAGAAATGGACC
CHS	Atru.chr6.2711	TCAAGCGCATGTGTGACAAA	TGCCTAGCTTTGGTACCTCC
UGT75C1	Atru.chr4.1539	CACCTACTCCACAACCGTCT	CGGACATGTACTGCTTCACG
CYP75B1	Atru.chr9.401	TTGGACGGAGATTGTTCGGA	TCCCATTACCAGCGTTGACT
Actin	Reference	CTCCCTTTATGCCAGTGGTC	TAGCATGAGGGAGGGAATAGC
β-tubulin	Reference	CTCGCTAACCCGCCTAAACA	ATGTCAAGTCCAGCGTGTGT

Table S5. The secondary structure of proteins encoded by candidate genes.

Name	ID	α-helix(%)	β-turn(%)	Extended strand(%)	Random coil(%)
F3H	Atru.chr3.799	35.61	7.19	15.83	41.37
FLS	Atru.chr13.1396	35.24	5.12	17.17	42.47
ANS	Atru.chr9.1498	34.34	3.77	15.85	46.04
LAR	Atru.chr11.1351	39.24	6.40	13.66	40.70
DFR	Atru.chr13.438	41.38	4.98	11.11	42.53
CHS	Atru.chr6.2711	41.73	5.09	14.50	38.68
UGT75C1	Atru.chr4.1539	41.53	5.93	14.62	37.92
CYP75B1	Atru.chr9.401	49.13	10.44	3.87	36.56

Table S6. Transcription factors affecting leaf colour.

Family	ID	PFAM	Pathway	Regulation
NAC	Atru.chr12.582	Chalcone	Map00941	UP
MYB	Atru.chr2.2623	COX15-CtaA	Map00860	UP
	Atru.chr2.2696	COX15-CtaA	Map00860	Down

Table S7. Structural domains of proteins encoded by candidate genes.

Name	ID	Number of Amino Acid	Domain ID	Start position	End position
F3H	Atru.chr3.799	279	PF14226	38	148
			PF03171	194	272
FLS	Atru.chr13.1396	332	PF14226	40	147
			PF03171	198	293
ANS	Atru.chr9.1498	265	PF14226	52	165
			PF03171	217	254
LAR	Atru.chr11.1351	344	PF05368	14	249
DFR	Atru.chr13.438	261	PF01370	6	163
CHS	Atru.chr6.2711	393	PF02797	5	228
			PF00195	238	388
UGT75C1	Atru.chr4.1539	472	PF00201	5	466
CYP75B1	Atru.chr9.401	517	PF00067	34	493
